# Dose-dependent perinatal risks of clomiphene citrate in US IVF cycles: a national cohort study, 2004–2021

**DOI:** 10.1136/bmjopen-2025-115285

**Published:** 2026-08-07

**Authors:** Sheree Boulet, Yujia Zhang, Dmitry Kissin, Michael Davies

**Affiliations:** 1 Department of Obstetrics and Gynecology, Boston University Chobanian and Avedisian School of Medicine, Boston, Massachusetts, USA; 2 Division of Reproductive Health, Centers for Disease Control and Prevention, Atlanta, Georgia, USA; 3 Robinson Research Institute and School of Biomedicine, University of Adelaide, Adelaide, South Australia, Australia

**Keywords:** in vitro fertilization, ovulation induction, embryo transfer, clomiphene citrate, adverse birth outcomes

## Abstract

**Abstract:**

**Objectives:**

The relationship between clomiphene citrate (CC) dosing regimens and adverse reproductive outcomes has not been fully elucidated, both with and without use of in vitro fertilisation (IVF). We aimed to study the association between cumulative CC dose and perinatal outcomes among fresh autologous IVF cycles.

**Design:**

Retrospective cohort study.

**Setting:**

We included all fresh autologous IVF embryo transfer cycles performed in the US fertility clinics during 2004–2021 using CC for ovulation induction. We used robust Poisson regression models to estimate adjusted risk ratios (aRRs) and 95% CIs for associations between four categories of CC dose (<500 mg, 500–749 mg, 750–999 mg and ≥1000 mg) and perinatal outcomes of interest.

**Participants:**

21 004 fresh autologous embryo transfer cycles using CC.

**Primary outcome measures:**

The primary outcome measures included biochemical pregnancy, clinical pregnancy, spontaneous abortion, stillbirth, live birth, multiple birth and preterm delivery.

**Results:**

Among fresh autologous embryo transfer cycles using CC, 21.3% used <500 mg, 67.8% used 500–749 mg, 7.2% used 750–999 mg and 3.7% used ≥1000 mg. After adjustment, rates of spontaneous abortion were higher for patients using 500–749 mg (11.8%; aRR, 1.12; 95% CI 1.02 to 1.25) and 750–999 mg of CC (14.4%; aRR, 1.38; 95% CI 1.18 to 1.62) than for those using <500 mg (10.3%). The ≥1000 mg group had a three-fold higher rate of stillbirth than the <500 mg group, but these estimates were imprecise (0.7% vs 0.2%; aRR, 3.31; 95% CI 0.88 to 12.40). Multiple birth risk was significantly higher among all CC dosage categories compared with the <500 mg group.

**Conclusion:**

Our findings extend previous findings on the association between CC exposure and adverse perinatal outcomes by demonstrating a dose-dependent relationship.

**STRENGTHS AND LIMITATIONS OF THIS STUDY**

- Cycles using clomiphene citrate comprised only 3% of all fresh, autologous in vitro fertilisation cycles during the study period.
- Some of the outcomes assessed in this observational study, including stillbirth, were rare and estimates were imprecise.
- We did not have access to information on clinical measures of ovarian reserve, use of other medications, endometrial thickness and embryo stage at transfer.

## Introduction

 Clomiphene citrate (CC) is often used as a first line treatment of anovulatory infertility to promote follicular development.^1^ It is also sometimes used during in vitro fertilisation (IVF) stimulation protocols to reduce the burden of hormonal injections and stimulation costs.^2^ CC can be used in place of follicle-stimulating hormone (FSH) in modified natural-cycle IVF, alongside FSH in mild ovarian stimulation protocols or concurrently with FSH in a costimulation protocol (conventional IVF).^3^ As a selective oestrogen receptor modulator (SERM), CC has close structural and functional similarities to tamoxifen and triparanol and some similarity to diethylstilbestrol.^4^ A common feature of SERMs is oestrogen antagonist or agonist effects, which vary by tissue and target organ.^5^ In the uterus, SERMs exhibit agonist activity and have been associated with increased risk for endometrial cancer among premenopausal and postmenopausal women taking tamoxifen for breast cancer treatment or prevention.^6 7^ Among women undergoing infertility treatment, CC use has also been identified as a potential risk factor for the development of thyroid and endometrial cancer, particularly for women exposed to higher doses or with extended use of 12 months or more.^8–11^

Despite global use of CC for ovulation induction alone or in combination with IVF, data from controlled studies on adverse reproductive events associated with CC use are limited but demonstrate consistent evidence of increased risk for multiple pregnancy, miscarriage, prematurity and birth defects.^1 12–15^ However, the relationship between dosing regimens and adverse outcomes remains understudied. Results from a recent study in mice suggest a dose-response association between CC and adverse reproductive outcomes, including declining pregnancy rates and increasing risks for fetal growth restriction, fetal loss and developmental anomalies in a dose-dependent manner.^16^ Moreover, the adverse effects were seen at doses that are equivalent to 50–100 mg in humans, a level that is consistent with contemporary practices for ovulation induction in women. Currently, the typical starting dose of CC for IVF is 50 mg per day for 5 consecutive days (starting on day 2–5 of the menstrual cycle or a random start for anovulatory infertility), resulting in a total cumulative dose of 250 mg.^1 17^ For women who do not respond to lower doses of CC, the dosage may be increased by 50 mg in subsequent cycles, up to a maximum of 250 mg, for a total cumulative dose of 1250 mg.^1^

CC competes with oestrogen receptor binding sites in the hypothalamus and pituitary and ultimately depletes oestrogen receptor concentrations by interfering with the normal process of replenishment.^1^ CC also has a high binding affinity, which contributes to slower rates of excretion and higher rates of accumulation of the drug both within the 5-day course and across treatment cycles.^13 18 19^ As a result, the routine practice of repeated and increasing doses during infertility treatments may pose an additional risk. To date, the risk of cumulative dose has not been assessed outside of animal studies. The aim of the current study was to estimate associations between total CC dose and perinatal outcomes of fresh autologous IVF cycles in the USA between 2004 and 2021. We hypothesised that rates of adverse outcomes would not increase with concomitant increases in total CC dose.

## Materials and methods

We used data from the National Assisted Reproductive Technology (ART) Surveillance System (NASS), a federally mandated surveillance system that collects information on all ART cycles performed in the United States.^20^ NASS defines ART cycles as fertility treatments in which human eggs or embryos are handled for the purpose of establishing a pregnancy. Although all clinics performing ART procedures are required to report information on patient and treatment characteristics as well as pregnancy outcomes to NASS, about 10% of clinics each year do not provide data.^20^ As those clinics tend to be small, NASS data represent an estimated 98% of ART cycles performed in the USA.^20^ A random sample of 7–10% of reporting clinics is selected each year for validation, and data reported to NASS are compared with medical records. Discrepancy rates for most fields are <5%, with some variation across data collection years. This study was approved by the institutional review board at the Centers for Disease Control and Prevention (#2238).

Because information on CC dose was not consistently collected for frozen embryo cycles across the study period, we restricted our analysis to all fresh IVF cycles performed between 2004 and 2021 in which CC was used for ovulation induction. NASS collects information on total CC dosage (in mg) that a patient received during an IVF cycle. Due to small numbers of donor oocyte cycles with reported CC dose, we further restricted the study population to autologous oocyte cycles. We considered total CC doses >2000 mg to be outliers and further excluded those cycles from analysis. We used linear regression to test for trends in annual mean total clomiphene dose over the study period. We created 4 categories of total clomiphene dosage, based on the distribution of the data, which was predominated by 500 mg. Given that CC is typically dispensed in packages of 10 tablets of 50 mg, we selected the 25th (500 mg), 85th (750 mg) and 95th (1000 mg) percentiles as the thresholds for the dosage categories.

The outcomes of interest were biochemical pregnancy (positive serum pregnancy test without ultrasound confirmation of a gestational sac within the uterus and without diagnosis of an ectopic pregnancy), clinical pregnancy (ultrasound-confirmed gestational sac within the uterus or the documented occurrence of a pregnancy outcome), spontaneous abortion (clinical pregnancy ending in spontaneous loss of the entire pregnancy prior to completion of 20 weeks of gestation or 18 weeks from the date of embryo transfer), stillbirth, live birth, multiple birth and preterm delivery (<37 weeks gestation). Because preterm delivery risk is higher for multiple births, we also calculated rates of preterm delivery among singleton births. All outcomes were assessed for fresh autologous transfers except multiple birth and preterm delivery, which were assessed for live births resulting from fresh autologous transfers.

We compared distributions of cycle and patient characteristics for the four dosage categories. Because information on race and ethnicity and/or body mass index (BMI) were missing for >30% of the cycles, we used multiple imputation to derive values for the missing data. Race and ethnicity were imputed at the patient level using methods described elsewhere.^21^ Briefly, the imputation models used 32 variables found to be associated with race and ethnicity, as well as patient’s state of residence and Census data reflecting the distribution of race and ethnicity in the patient’s residential zip code. We generated 20 datasets for analysis. BMI was imputed at the cycle level, which allowed BMI to vary between cycles for the same patient. We used linear regression models with 21 covariates to impute BMI as a continuous variable and created 20 multiply imputed datasets. After imputation, outliers (BMI values <12 or >70 kg/m^2^) were removed and replaced with the patient’s mean imputed values. We calculated the distribution of race and ethnicity and BMI categories (<18.5 kg/m^2^, 18.5–24.9 kg/m^2^, 25.0–29.9 kg/m^2^ and ≥30 kg/m^2^) across the four dosage categories for each of the 20 imputed datasets and pooled the resulting parameter estimates and standard errors to create combined estimates.

We used Cochran-Armitage tests to assess trends in crude rates of outcomes across the CC dose categories. Using robust Poisson regression models with generalised estimating equations to account for clustering by clinic, we estimated adjusted risk ratios (aRRs) and 95% CIs for the association between clomiphene dose categories and the perinatal outcomes of interest described above. The models adjusted for factors selected a priori based on current knowledge and expert opinion and included age at cycle start, parity, number of prior ART cycles, infertility diagnosis, race and ethnicity and BMI. We estimated the regression models separately for each of the 20 imputed datasets and pooled the parameter estimates and SEs to generate combined effect estimates and 95% CIs. As a sensitivity analysis, we also estimated associations between CC dose and rates of clinical pregnancy, spontaneous abortion, stillbirth and live birth among singleton gestations only. We used SAS V.9.4 (SAS Institute, North Carolina, USA) and R V.4.3.2 (R Core Team, Austria) for analysis. P values <0.05 were considered statistically significant.

### Patient and public involvement

Patients were not involved in the design, conduct, reporting or dissemination plans of our research.

## Results

**Figure 1:**
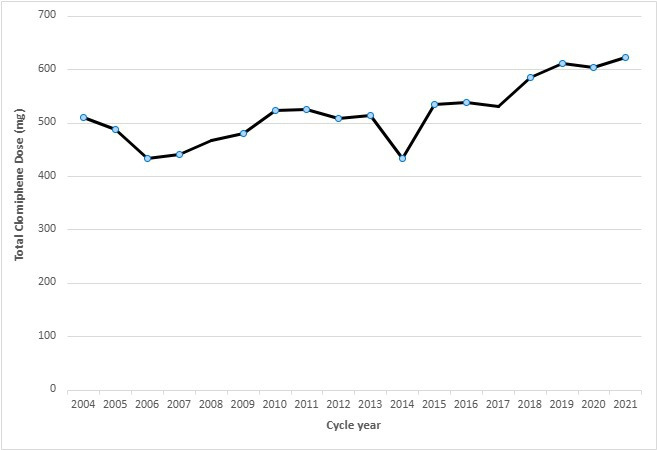
Trends in mean total clomiphene dosage among fresh autologous in vitro fertilisation cycles, 2004–2021. P for linear trend <0.0001.

There were 1 698 813 fresh cycles from 2000 to 2021 (141 941 donor and 1 556 872 autologous). Among the autologous cycles, 47 705 (3.1%) reported information on total CC dosage. We excluded 280 cycles with total clomiphene dose >2000 mg. In the resulting sample of 47 425 fresh autologous cycles, the mean total CC dose increased from 510.5 mg in 2004 to 621.8 mg in 2021 (β=9.86; SE 0.22; p<0.0001) (figure 1). The distribution of total CC dose is shown in online supplemental figure S1 (available online).

After restricting the analysis to cycles in which at least one embryo was transferred, the final dataset had 21 004 fresh autologous embryo transfer cycles. The median total CC dose was 500 mg, with 4476 patients (21.3%) using <500 mg, 14 241 (67.8%) using 500–749 mg, 1517 (7.2%) using 750–999 mg and 770 (3.7%) using 1000 mg or more (table 1). Patients using higher doses of CC tended to be younger (<35 years), identify as non-Hispanic white or black, be multiparous, have two or more prior ART cycles and have a BMI of 30 kg/m^2^ or higher. Diminished ovarian reserve was the most common infertility diagnosis regardless of CC dosage category.

**Table 1:** Characteristics of fresh autologous in vitro fertilisation embryo transfer cycles by categories of total clomiphene dose, 2004–2021

	Total clomiphene citrate dose			
	**<500** mg **N=**4476	**500–749** mg **N=**14 241	**750–999** mg **N=**1517	**≥1000** mg **N=770**
**Characteristic**	**N (%**)	**N (%**)	**N (%**)	**N (%**)
Patient age (years)				
<30	295 (6.6)	791 (5.6)	133 (8.8)	71 (9.2)
30–34	784 (17.5)	2575 (18.1)	299 (19.7)	177 (23.0)
35–39	1367 (19.6)	4885 (32.3)	511 (33.7)	231 (30.0)
≥40	2030 (45.4)	5990 (42.1)	574 (37.8)	291 (37.8)
Race and ethnicity*				
Non-Hispanic white	2262 (50.5)	8988 (63.1)	974 (64.2)	480 (62.3)
Non-Hispanic black	422 (9.4)	1194 (8.4)	140 (9.3)	89 (11.6)
Asian	1283 (28.7)	2446 (17.2)	212 (14.0)	105 (13.6)
Hispanic	463 (10.3)	1435 (10.1)	168 (11.1)	84 (10.8)
Native Hawaiian/Pacific Islander	12 (0.3)	38 (0.23)	6 (0.4)	1 (0.2)
American Indian/Alaska Native	20 (0.4)	50 (0.4)	6 (0.4)	10 (1.3)
Two or more races	15 (0.3)	90 (0.6)	10 (0.6)	2 (0.3)
Infertility diagnosis				
Tubal factor	680 (15.2)	2380 (16.7)	256 (16.9)	109 (14.2)
Endometriosis	310 (6.9)	1238 (8.7)	126 (8.3)	72 (9.4)
Uterine factor	252 (5.6)	795 (5.6)	59 (3.9)	38 (4.9)
Ovulatory disorder	437 (9.8)	1538 (10.8)	168 (11.1)	97 (12.6)
Diminished ovarian reserve	2118 (47.3)	7421 (52.1)	712 (46.9)	352 (45.7)
Male factor	935 (20.9)	3956 (27.8)	430 (28.4)	202 (26.2)
Unexplained	647 (14.5)	1522 (10.7)	186 (12.3)	90 (11.7)
Other	698 (15.6)	1665 (11.7)	181 (11.9)	96 (12.5)
Parity**†**				
0	3198 (71.7)	10 072 (70.9)	1019 (67.4)	506 (65.7)
1	924 (20.7)	3128 (22.0)	339 (7.4)	185 (24.0)
≥2	337 (7.6)	1012 (7.1)	155 (10.2)	79 (10.3)
Prior ART cycles				
0	1728 (38.6)	4542 (31.9)	552 (36.4)	219 (28.4)
1	987 (22.1)	3449 (24.2)	386 (25.4)	223 (28.9)
≥2	1761 (39.3)	6250 (43.9)	579 (38.2)	328 (42.6)
BMI (kg/m^2^)*				
<18.5	184 (4.1)	476 (3.3)	40 (2.6)	23 (3.4)
18.5–24.9	2214 (49.5)	7210 (50.6)	643 (42.4)	302 (39.2)
25.0–29.9	1379 (30.8)	4168 (29.2)	447 (29.5)	235 (30.6)
≥30	1699 (15.6)	2388 (16.8)	387 (25.5)	209 (27.2)

Note: All associations significant at p<0.05 except ovulatory disorder (p=0.06).

* Values were imputed due to high percentage of missing data.

† Parity information was missing for 50 cycles.

ART, assisted reproductive technology; BMI, body mass index; kg, kilograms; m, metre; mg, milligrams.

The overall rate of biochemical pregnancy was 6.9% and did not vary markedly across the CC dosage categories (table 2). The rate of clinical pregnancy increased from 25.1% in the <500 mg group to 28.6% in the ≥1000 mg group (p<0.05), but the association was not statistically significant after adjustment. Rates of spontaneous abortion among patients using 500–749 mg, 750–999 mg and ≥1000 mg of CC were higher than the <500 mg group (11.8%, 14.4% and 12.5% vs 10.3%, respectively; p<0.05). After adjusting for age, parity, number of prior ART cycles, infertility diagnosis, race/ethnicity and BMI, use of 500–749 mg and 750–999 mg of CC was associated with increased risk for spontaneous abortion (aRR, 1.12; 95% CI 1.02 to 1.25 and aRR, 1.38; 95% CI 1.18 to 1.62, respectively). Stillbirth rates also increased incrementally across the CC dosage categories (p<0.05), with the highest rate occurring in the ≥1000 mg group (0.7%). However, the effect estimate was imprecise due to small sample size, and the CI included the null (aRR, 3.30; 95% CI 0.88 to 12.40). Live birth rates increased from 18.6% (<500 mg group) to 22.5% (≥1000 mg group), with no significant differences observed after adjusting for age, parity, number of prior ART cycles, infertility diagnosis, race/ethnicity and BMI. Among singleton gestations, use of 500–749 mg and 750–999 mg of CC was also associated with increased risk for spontaneous abortion (aRR, 1.32; 95% CI 1.07 to 1.62 and aRR, 1.47; 95% CI 1.08 to 2.00, respectively) (online supplemental table S1, available online). The number of stillbirths among singleton gestations was too small to reliably estimate associations.

**Table 2:** Association between categories of total clomiphene dosage and perinatal outcomes in 21 004 fresh autologous in vitro fertilisation embryo transfer cycles, 2004–2021

	Total clomiphene citrate dose							
	**<500** mg **N=**4476		**500–749** mg **N=**14 241		**750–999** mg **N=**1517		**≥1000** mg **N=770**	
	N (%)	aRR* (95% CI)	N (%)	aRR* (95% CI)	N (%)	aRR* (95% CI)	N (%)	aRR* (95% CI)
Biochemical pregnancy	305 (6.8)	1.00	996 (7.0)	1.00 (0.87 to 1.13)	101 (6.7)	0.97 (0.77 to 1.21)	53 (6.9)	0.98 (0.77 to 1.31)
Clinical pregnancy	1122 (25.1)	1.00	3881 (27.3)	1.06 (1.00 to 1.13)	440 (29.0)	1.06 (0.97 to 1.17)	220 (28.6)	1.05 (0.93 to 1.18)
Spontaneous abortion	463 (10.3)	1.00	1685 (11.8)	1.12 (1.02 to 1.25)	218 (14.4)	1.38 (1.18 to 1.62)	96 (12.5)	1.18 (0.95 to 1.47)
Stillbirth	8 (0.2)	1.00	41 (0.3)	1.57 (0.74 to 3.34)	6 (0.4)	1.99 (0.74 to 5.89)	5 (0.7)	3.31 (0.88 to 12.40)
Live birth	834 (18.6)	1.00	2823 (19.8)	1.03 (0.96 to 1.10)	324 (21.4)	1.02 (0.91 to 1.14)	173 (22.5)	1.08 (0.94 to 1.23)

Note: Cochran-Armitage test p<0.05 for trends in rates of clinical pregnancy, spontaneous abortion, stillbirth and live birth across dose categories.

* Adjusted for age, parity, number of prior ART cycles, infertility diagnosis, race/ethnicity (imputed) and body mass index (imputed).

aRR, adjusted risk ratio; ART, assisted reproductive technology; mg, milligrams.

Multiple birth risk increased incrementally across CC dosage categories compared with the <500 mg group (13.2%): 17.7%; aRR, 1.35; 95% CI 1.12 to 1.64 for the 500–749 mg group, 21.3%; aRR, 1.55; 95% CI 1.18 to 2.04 for the 750–999 mg group and 27.2%; aRR, 1.88; 95% CI 1.39 to 2.54 for the ≥1000 mg group. (table 3) Preterm delivery rates ranged from 17.2% in the <500 mg group to 20.2% in the ≥1000 mg group (p<0.05), but the effect estimates were not statistically significant after adjustment. When restricted to singleton births, rates of preterm delivery were lower overall and ranged from 10.7% in the 500–749 mg group to 15.9% in the ≥1000 mg group with no statistically significant associations observed.

**Table 3:** Association between categories of total clomiphene dosage and perinatal outcomes in 4154 live births resulting from fresh autologous in vitro fertilisation embryo transfer cycles, 2004–2021

	Total clomiphene citrate dose							
	**<500** mg **N=834**		**500–749** mg **N=**2823		**750–999** mg **N=324**		**≥1000** mg **N=173**	
	N (%)	aRR* (95% CI)	N (%)	aRR* (95% CI)	N (%)	aRR* (95% CI)	N (%)	aRR* (95% CI)
Multiple birth	110 (13.2)	1.00	499 (17.7)	1.35 (1.12 to 1.64)	69 (21.3)	1.55 (1.18 to 2.04)	47 (27.2)	1.88 (1.39 to 2.54)
Preterm delivery†	143 (17.2)	1.00	556 (19.7)	1.17 (0.98 to 1.38)	62 (19.2)	1.13 (0.86 to 1.48)	35 (20.2)	1.15 (0.82 to 1.61)
Preterm delivery (singleton births)‡	86 (11.9)	1.00	249 (10.7)	0.91 (0.71 to 1.15)	30 (11.8)	1.01 (0.68 to 1.50)	20 (15.9)	1.36 (0.86 to 2.14)

Note: Cochran-Armitage test p<0.05 for trends in rates of multiple birth and preterm delivery (total) across dose categories.

* Adjusted for age, parity, number of prior ART cycles, infertility diagnosis, race/ethnicity (imputed) and body mass index (imputed).

† Gestational age data was missing for four births.

‡ Among singleton live births with the following denominators: 724 (<500 g), 2322 (500–749 mg), 254 (750–999 mg) and 126 (≥1000 mg).

aRR, adjusted risk ratio; ART, assisted reproductive technology; mg, milligrams.

## Discussion

The findings from this study of national data indicate that total CC dose in fresh autologous IVF cycles performed in the USA increased from 2004 to 2021. This secular increase in cumulative dose may reflect either a shift to a more aggressive treatment strategy or changes in population body mass, which is robustly related to effective dose.^13^ We also found that exposure to CC doses at or above the median of 500 mg was associated with increased risk for spontaneous abortion, stillbirth and multiple birth. Although some effect estimates were imprecise because the outcomes of interest were rare (for stillbirth in particular), the combined findings of elevated risks for adverse perinatal outcomes with higher doses of CC and a linear increase in average total CC dose suggest that continued monitoring of CC use in IVF may be warranted.

We observed a dose-response relationship between increasing CC dose and multiple birth risk. This finding differs from a prior study where data on CC dose was measured in the 90 days before conception,^13^ not across treatment courses where bioaccumulation may become more relevant. It provides further evidence that CC may be a causal driver of iatrogenic multiple gestation in the context of IVF. The mechanism underlying elevated rates of multiple gestation among IVF cycles using CC for ovulation induction is due to direct increases in FSH and luteinizing hormone (LH), and potentially increased sensitivity of granulosa cells to gonadotropins^13^ and processes related to increased risk for iatrogenic monozygotic twins.^22^ It is clinically relevant because of the additional developmental risks, including attention-deficit/hyperactivity disorder and autism spectrum disorder, compared with dizygotic twins and singletons.^23 24^ While multiple pregnancy has attracted much attention within the IVF literature, with recent temporal reductions due to the adoption of single embryo transfer in routine IVF cycles,^25 26^ the multiple birth rate observed in the current study remains concerning. Multiple gestation pregnancies are considered high risk, with increased rates of hypertensive disorders of pregnancy, caesarean delivery and maternal death compared with singletons.^27^ This has been shown elsewhere where twinning from CC now exceeds that from IVF.^28^ For the child, there is an increased risk of prematurity and prematurity-related diseases, including stroke, cerebral palsy, admission to neonatal intensive care, growth restriction and long-term cognitive disability mediated through prematurity and cerebral accident.^28–31^ The long-term consequences of multiple pregnancy remain an understudied area, although the imperative for doing so is indicated by the total costs of care for the first year of life for higher order pregnancies compared with singleton or twin pregnancies.^32^

We also identified a dose-response association for miscarriage and stillbirth, culminating in a threefold increased risk for stillbirth among patients using >1000 mg of CC relative to those using <500 mg, although estimates for stillbirth were imprecise due to small numbers. This study is consistent with another human cohort study that identified a significant increase in perinatal death (the summing of stillbirth and neonatal death) from CC administered proximal to a conception window.^33^ It extends the findings by demonstrating a dose-response relationship, rather than a periconceptional timing of exposure, for related outcomes. The dose-response relationship reported here also replicates a recent animal study designed to evaluate CC, which also demonstrated increased rates of pregnancy loss in a dose-dependent manner.^16^ Collectively, these three studies triangulate the relationship between CC and adverse pregnancy outcomes by relating the elements of temporal proximity of exposure to conception, biological plausibility in an experimental mouse model and dose-response relationship with cumulative dose.

With regards to potential mechanisms for adverse outcomes following exposure to CC and implications for further research, it is established that in addition to twinning, ovulation induction results in a thinner endometrium due to anti-oestrogenic effects of CC, resulting in impaired vascularisation, poor implantation, impaired fetal development and potential fetal arrest.^34^

Our findings are subject to several limitations. Our most significant limitation is the availability of CC dosage data for only 3.1% of eligible cycles, raising concerns about selection bias. Cycles with dosage data may represent patients with more complex treatment histories or clinics with more detailed documentation practices. If these cycles are systematically different from those without dosage data, our estimates may not be representative of the broader population undergoing IVF with CC. Notably, our overall rates of spontaneous abortion, stillbirth and live birth were lower than national rates among the general IVF population in 2021 (11.7 vs 15.5%, 0.3% vs 0.5% and 20% vs 49%, respectively).^20^ There is also variability in medication outcomes due to patient factors, as the pharmacokinetics are influenced by drug metabolism and body mass, such that some women may require escalating doses to achieve an effective dose.^13^ The drug–patient interaction should be examined further to obtain a tailored minimum effective dose. In addition, our study was restricted to fresh cycles, and we could not evaluate outcomes in frozen embryo transfer cycles, where rates of adverse perinatal outcomes such as pregnancy loss may be lower overall.^35^ We were also unable to evaluate neonatal death, which may be higher in pregnancies following CC.^33^ Our study was underpowered to detect statistically significant associations for rare outcomes such as stillbirth. The wide CIs for these estimates reflect this limitation and indicate the need for larger studies or pooled analyses. Our study was limited to CC used in combination with IVF and does not account for the contribution of IVF itself, variations in CC stimulation protocols or use of other medications, including FSH, to the observed outcomes. A recent randomised trial found no difference in miscarriage or live birth rates between standard and increased FSH dosing among anticipated poor responders undergoing IVF, suggesting that differences in concurrent FSH use were unlikely to meaningfully influence our findings.^36^ We do not report the outcome of ovarian hyperstimulation syndrome, which was too rare to reliably estimate. We imputed data on race, ethnicity and BMI, which may introduce bias. In addition, we did not have access to information on number of oocytes retrieved, clinical measures of ovarian reserve, endometrial thickness, embryo stage of transfer and number of embryos transferred and were unable to evaluate their impact on the observed outcomes. However, some of these factors may be on the causal pathway and thus should not be controlled for. Finally, given the observational nature of our study, our results are also affected by unmeasured confounding and bias.

This study adds to the limited evidence on individual response to CC, where there is some evidence of both a threshold effect and a dose-response relationship. Most patients respond rapidly to CC, in contrast to the subgroup recognised as ‘poor responders’, indicated in this study by relatively high cumulative doses of CC but without a rise in birth rate. We also found that, as cumulative dose increases, birth rate plateaus, but rates of multiple birth steadily increase. The outcome is an escalation in risk without benefit to the patient group, highlighting the need for identifying sources of variability in patient response to enable individual dosing strategies. Additional studies of CC metabolism within and across cycles are needed to identify pathways for individual variability in response to stimulation and its effects on fetal development and risk of multiple gestations. Further research with a larger sample size is also warranted to confirm the association between CC dosage and risk of stillbirth. Additional factors for future examination include the established relationship between maternal body mass and responsiveness to clomiphene, but at scale^13^; and the interaction with genetic polymorphisms that alter the effectiveness of liver enzyme CYP2D6 which is essential for effective clomiphene metabolism.^37^

## Supplementary material

10.1136/bmjopen-2025-115285 online supplemental file 1

10.1136/bmjopen-2025-115285 online supplemental file 2

## Data Availability

Data may be obtained from a third party and are not publicly available.
