# Supplementary material for: Dose-dependent perinatal risks of clomiphene citrate in US IVF cycles: a national cohort study, 2004–2021
Source: BMJ Open. 2026 Aug 7;16(8):e115285. doi: 10.1136/bmjopen-2025-115285 (PMC13475558; doi:10.1136/bmjopen-2025-115285)
Supplement: Supplementary file 1 [file bmjopen-2025-115285.reviewer_comments.pdf]

PEER REVIEW HISTORY

BMJ Open publishes all reviews undertaken for accepted manuscripts. Reviewers are asked to complete a checklist review form and are provided with free text boxes to elaborate on their assessment. These free text comments are reproduced below.

ARTICLE DETAILS

Title (Provisional)

Dose-Dependent Perinatal Risks of Clomiphene Citrate in U.S. IVF Cycles: A National Cohort Study, 2004-2021

Authors

Boulet, Sheree; Zhang, Yujia; Kissin, Dmitry; Davies, Michael

VERSION 1 - REVIEW

**Reviewer**	**1**
**Name**	**Thanaboonyawat, Isarin**
**Affiliation**	**Mahidol University, Obstetrics and gynecology**
**Date**	**15-Mar-2026**
**COI**	**None**

This is a retrospective study enrolled in a certain number of cases. The manuscript was well written; however, some points need to be clarified.

Introduction

Line 78-80: It is better to state clearly that the SERM mentioned was tamoxifem to avoid confusion.

Line 80-82: When discussing the connection between thyroid cancer and clomiphene citrate, it is more appropriate to cite the original article rather than relying mainly on guidelines.

Line 101; reference 4 is not associated with CC.

Methods

Is clomiphene citrate (CC) administered as a monotherapy, or in combination with other medications such as letrozole or gonadotropins during the IVF cycles included in the study? If combination therapies are present, the authors should account for potential confounding effects from additional medications on outcomes such as birth defect incidence or pregnancy rate.

Was luteal support provided, and if so, what agents were used? The choice of medication for luteal phase support is another important confounding variable; for instance,

dydrogesterone and other progesterone derivatives have been reported to correlate with specific birth defects.

The stimulation protocol should be specified. Did patients receive CC for only 5 days, or was it administered for more than 5 days if they did not respond during the initial round of CC stimulation? Is duration a factor affecting the results?

Results

Line 198: should be Table 2

Discussion

Line 221: Please define "treatment course" in the methods. Reference 12 considers cycles exposed to CC within 90 days before conception. What is the exposure period defined in this study? Any differences when compared to reference 12?

There are many factors that affect pregnancy rates or complications that were not collected. The results concluded in this study can hardly suggest that the cumulative effects of CC affect the still birth or other complications.

**Reviewer**	**2**
**Name**	**Jones, Ann**
**Affiliation**	**University of Toronto**
**Date**	**15-Jun-2026**
**COI**	**None**

"Dose-Dependent Perinatal Risks of Clomiphene Citrate in U.S. IVF Cycles: A

4 National Cohort Study, 2004-2021"

Summary:

This is a well written manuscript assessing the impact of clomiphene citrate dose on adverse pregnancy outcomes in fresh autologous IVF cycles

Abstract:

- Clearly written. line 47 there is a closed bracket instead of a comma

Introduction:

- Well written. No concerns. Could consider adding a sentence about how clomiphene is being used in IVF cycles (ie instead of FSH, overlapping with FSH in a minimal stimulation protocol or administered concurrently with FSH in a co-stimulation protocol)

Methods:

- Clearly described methods

- What percentage of clinics across the USA have data reported in NASS? Is there mandatory reporting to NASS or could there be lots of missing clinic data?

- I wondered why daily or total FSH dose was not included as a potential variable impacting outcome which is not discussed in the methods at all, nor the outcome of ovarian hyperstimulation syndrome which could also potentially be a variable that could impact adverse pregnancy outcomes.

Results:

- page 11, line 189, please add what the results for spontaneous abortion were adjusted for.

- page 11, line 196, please add what the results of live birth were adjusted for

- No data on whether FSH was given in conjunction with CC or not in table 1 or in results and similarly do data on FSH dose or number of oocytes retrieved

Conclusion:

- page 13, line 274 I think should read "…factors may be on the causal pathway and thus should be controlled for"

- No discussion regarding lack of data on FSH co-administration or lack therefore and dosing and risk for OHSS which may impact fresh IVF cycle outcomes – recommend adding this to the discussion. Were these min-stim protocols, co-stim protocols or isolated CC only stim protocols and how might this impact outcomes

- Could add a bit more to the discussion regarding BMI as escalating doses were likely used in higher BMI patients due to lack of response at lower doses and so it may not be possible to give lower doses of clomiphene to some of these patients, and this patient population are at higher risk for adverse pregnancy outcomes just based on BMI alone. Do the authors have suggestions on how to address this problem? Should these patients just be receiving FSH alone and not include clomiphene if they are at such high risk of adverse pregnancy outcomes?

- It would be nice to include what the rate of miscarriage and stillbirth (as well as live birth rate) was in fresh autologous IVF cycles without clomiphene citrate in the same database over the same time period to know how different this patient population is compared to the general IVF patient population.

VERSION 1 - AUTHOR RESPONSE

Response to reviewers

Reviewer comment	Response	Revised text
**Reviewer 1**		
Introduction<br>Line 78-80: It is better to state clearly that the SERM mentioned was tamoxifem to avoid confusion.	We edited the sentence to clarify.	"In the uterus, SERMs exhibit agonist activity and have been associated with increased risk for endometrial cancer among pre- and postmenopausal women taking tamoxifen for breast cancer

		treatment or prevention (6, 7)."
Line 80-82: When discussing the connection between thyroid cancer and clomiphene citrate, it is more appropriate to cite the original article rather than relying mainly on guidelines.	Quite correct. The literature is mixed, so we have added 3 additional citations for systematic reviews and a meta-analysis for the associations between clomiphene and cancers.	Additional references (9-11) were included.
Line 101; reference 4 is not associated with CC.	Our apologies. The Dickey reference (ref 9) was intended here as this shows the accumulation of clomiphene across and between courses.	The correct reference (13) was included.
Methods<br>Is clomiphene citrate (CC) administered as a monotherapy, or in combination with other medications such as letrozole or gonadotropins during the IVF cycles included in the study? If combination therapies are present, the authors should account for potential confounding effects from additional medications on outcomes such as birth defect incidence or pregnancy rate.	The eligible population for this study included nearly all fertility clinics in the US (e.g., >450 clinics in 2021). Treatment practices vary across clinics and cannot be fully accounted for in this study. Information on other medications is limited in this national surveillance database. We added text to note this as limitation.	"Our study was limited to CC used in combination with IVF and does not account for the contribution of IVF itself, <u>variations in CC stimulation protocols</u>, or use of other medications, <u>including follicle-stimulating hormone</u>, to the observed outcomes."
Was luteal support provided, and if so, what agents were used? The choice of medication for luteal phase support is another important confounding variable; for instance, dydrogesterone and other progesterone derivatives have been reported to correlate with specific birth defects.	Please see above explanation. We added text to note this as limitation.	"Our study was limited to CC used in combination with IVF and does not account for the contribution of IVF itself, <u>variations in CC stimulation protocols</u>, or use of other medications, <u>including follicle-stimulating hormone</u>, to the observed outcomes."
The stimulation protocol should be specified. Did patients receive CC for	Clomiphene is typically administered as a course of 5 days starting on day 2-5. This is	"Currently, the typical starting dose of CC for IVF is 50 mg per day for

only 5 days, or was it administered for more than 5 days if they did not respond during the initial round of CC stimulation? Is duration a factor affecting the results?	repeated monthly until pregnancy is detected.<br>Most women who respond do so with a single 5-day course. The reason for repeated courses is variable, and may be due to resistance to clomiphene, or factors related to infertility, or factors related to the associated IVF protocol. A number of these are recorded (e.g. age) and are included in the models. We do not know of an unmeasured confounder that can display a dose-response relationship with the outcome of the magnitude observed.<br><br>However, the database does not include information on individual CC stimulation protocols, only cumulative dose for a given cycle. As noted in CDC's 2023 Federal Register notice (2023-26137 (88 FR 83131), CC dosage regimens in US fertility clinics typically follow established guidelines and do not show significant variability.<br><br>Supplemental Figure 1 indicates that the vast majority of cycles received 500mg of CC, which is consistent with current guidelines and suggests that most patients received 100mg/day for 5 days. However, we agree this factor should be considered and added variations in stimulation protocols as a limitation.	5 consecutive days (starting on day 2-5 of the menstrual cycle or a random start for anovulatory infertility), resulting in a total cumulative dose of 250 mg (1, 17)."<br><br>"Our study was limited to CC used in combination with IVF and does not account for the contribution of IVF itself, variations in CC stimulation protocols, or use of other medications, including follicle-stimulating hormone, to the observed outcomes."
Results<br>Line 198: should be Table 2	The effect estimates mentioned in the text are reported in Supplemental Table 1. We reference Table 2 earlier in the paragraph.	
Discussion<br>Line 221: Please define "treatment course" in the	We clarified how the cited study measured CC and provided additional information on how CC	"This finding differs from a prior study where data on CC dose was measured

methods. Reference 12 considers cycles exposed to CC within 90 days before conception. What is the exposure period defined in this study? Any differences when compared to reference 12?	is typically administered in our population.<br><br>Per CDC's reporting guidelines, the exposure period for fresh embryo transfer cycles begins with the first day of clomiphene usage.	in the 90 days before conception (13), not across treatment courses where bioaccumulation may become more relevant."<br><br>"Currently, the typical starting dose of CC for IVF is 50 mg per day for 5 consecutive days (starting on day 2-5 of the menstrual cycle or a random start for anovulatory infertility), resulting in a total cumulative dose of 250 mg (1, 17). For women who do not respond to lower doses of CC, the dosage may be increased by 50 mg in subsequent cycles, up to a maximum of 250 mg, for a total cumulative dose of 1,250 mg (1)."
There are many factors that affect pregnancy rates or complications that were not collected. The results concluded in this study can hardly suggest that the cumulative effects of CC affect the still birth or other complications.	We agree that these findings are subject to several limitations, as indicated in the manuscript, including limited power to detect associations with stillbirths. We state in the conclusions that: "Further research with a larger sample size is also warranted to confirm the association between CC dosage and risk of stillbirth."	
**Reviewer 2**		
Abstract:<br>- Clearly written. line 47 there is a closed bracket instead of a comma	Thank you. We corrected this typo.	
Introduction:<br>- Well written. No concerns. Could consider adding a sentence about how clomiphene is being used in IVF cycles (ie instead of FSH, overlapping with FSH in	Thank you. We added a sentence describing use of clomiphene in IVF cycles.	"CC can be used in place of follicle-stimulating hormone (FSH) in modified natural-cycle IVF, alongside FSH in mild ovarian stimulation protocols, or concurrently with FSH in a co-

a minimal stimulation protocol or administered concurrently with FSH in a co-stimulation protocol)		stimulation protocol (conventional IVF). (3)”
- What percentage of clinics across the USA have data reported in NASS? Is there mandatory reporting to NASS or could there be lots of missing clinic data?	Although reporting to NASS is mandatory, approximately 10% of clinics annually do not provide the required data. However, the non-reporting clinics are small, and it is estimated that NASS data covers about 98% of cycles in the US. We added this information to the methods.	“We used data from the National Assisted Reproductive Technology (ART) Surveillance System (NASS), a federally mandated surveillance system that collects information on all ART cycles performed in the United States (20). NASS defines ART cycles as fertility treatments in which human eggs or embryos are handled for the purpose of establishing a pregnancy. Although all clinics performing ART procedures are required to report information on patient and treatment characteristics as well as pregnancy outcomes to NASS, about 10% of clinics each year do not provide data. (20) As those clinics tend to be small, NASS data represent an estimated 98% of ART cycles performed in the U.S.”
- I wondered why daily or total FSH dose was not included as a potential variable impacting outcome which is not discussed in the methods at all, nor the outcome of ovarian hyperstimulation syndrome which could also potentially be a variable that could impact adverse pregnancy outcomes.	While we do not report FSH, we report (and control for) the diagnosis of diminished ovarian reserve, which is typically defined as FSH>10 mIU/mL. We added this as a limitation.<br><br>OHSS is a rare outcome, with data from a 2020 study using NASS data indicating that only 5 per 1,000 cycles in 2016-2015 were affected (doi: 10.1016/j.fertnstert.2020.04.004)	“Our study was limited to CC used in combination with IVF and does not account for the contribution of IVF itself, variations in CC stimulation protocols, or use of other medications, including FSH, to the observed outcomes. We do not report the outcome of ovarian hyperstimulation

	While we explored it as a potential outcome for the current study, the number of cases was too small to reliably estimate for this study.	syndrome, which was too rare to reliably estimate. ”<br><br>We also added the limitation to the bulleted list of strengths and limitations of the study (line 66).<br><br>• We did not have access to information on clinical measures of ovarian reserve, use of other medications, endometrial thickness, and embryo stage at transfer.
Results:<br>- page 11, line 189, please add what the results for spontaneous abortion were adjusted for.	We have added this information.	After adjusting for age, parity, number of prior ART cycles, infertility diagnosis, race/ethnicity, and BMI, use of 500–749 mg and 750–999 mg of CC was associated with increased risk for spontaneous abortion (aRR, 1.12; 95% CI, 1.02–1.25 and aRR, 1.38; 95% CI, 1.18–1.62, respectively).
- page 11, line 196, please add what the results of live birth were adjusted for	We added this information.	Live birth rates increased from 18.6% (<500 mg group) to 22.5% (≥1,000 mg group), with no significant differences observed after adjusting for age, parity, number of prior ART cycles, infertility diagnosis, race/ethnicity, and BMI.
- No data on whether FSH was given in conjunction with CC or	Noted: Please see comment above.	“Our study was limited to CC used in combination with IVF and does not account for the

not in table 1 or in results and<br>similarly do data on FSH dose or number of oocytes retrieved		contribution of IVF itself, variations in CC stimulation protocols, or use of other medications, including FSH, to the observed outcomes."
Conclusion:<br>- page 13, line 274 I think should read "…factors may be on the causal pathway and thus should be controlled for"	Factors on the causal pathway from an exposure to an outcome (also known as mediators) should NOT be controlled for as this would bias estimates.<br><br>doi: 10.1097/EDE.0b013e3181a819a1.	
- No discussion regarding lack of data on FSH co-administration or lack therefore and dosing<br>and risk for OHSS which may impact fresh IVF cycle outcomes – recommend adding this to the<br>discussion. Were these min-stim protocols, co-stim protocols or isolated CC only stim protocols and how might this impact outcomes	We do not have access to FSH data for this population. We added additional discussion of this to the limitations.	"Our study was limited to CC used in combination with IVF and does not account for the contribution of IVF itself, variations in CC stimulation protocols, or use of other medications, including FSH, to the observed outcomes. A recent randomized trial found no difference in miscarriage or live birth rates between standard and increased FSH dosing among anticipated poor responders undergoing IVF, suggesting that differences in concurrent FSH use were unlikely to meaningfully influence our findings. (36)"
- Could add a bit more to the discussion regarding BMI as escalating doses were likely used in higher BMI patients due to lack of response at lower doses and so it may not be possible to give lower doses of clomiphene to some of these patients, and this	We agree and have added this as a limitation.<br><br>Regrettably, examining gene/body mass interactions is beyond the scope of the present study as genetic polymorphisms for clomiphene are not routinely collected.	"There is also variability in medication outcomes due to patient factors, as the pharmacokinetics are influenced by drug metabolism and body mass, such that some women may require escalating doses to achieve an effective dose. The drug-patient

patient population are at higher risk for adverse pregnancy outcomes just based on BMI alone.<br><br>Do the authors have suggestions on how to address this problem? Should these patients just be receiving FSH alone and not include clomiphene if they are at such high risk of adverse pregnancy outcomes?	However, this work may have been done for tamoxifen where ascertaining a timely and effective dose may be critical for survival.	interaction should be examined further to obtain a tailored minimum effective dose."
- It would be nice to include what the rate of miscarriage and stillbirth (as well as live birth rate) was in fresh autologous IVF cycles without clomiphene citrate in the same database over the same time period to know how different this patient population is compared to the general IVF patient population	We added this information based on published data.	"Notably, our overall rates of spontaneous abortion, stillbirth, and livebirth were lower than national rates among the general IVF population in 2021 (11.7 vs 15.5%, 0.3% vs 0.5%, and 20% vs 49%, respectively) (20)."
