# Supplementary material for: Dose-dependent perinatal risks of clomiphene citrate in US IVF cycles: a national cohort study, 2004–2021
Source: BMJ Open. 2026 Aug 7;16(8):e115285. doi: 10.1136/bmjopen-2025-115285 (PMC13475558; doi:10.1136/bmjopen-2025-115285)
Supplement: Supplementary file 2 [file bmjopen-16-8-s002.docx]

**Supplemental Figure 1.**

Legend: Distribution of total clomiphene dosage in 47,425 fresh in vitro fertilization cycles, 2004–2021

**Supplemental Table 1. Association between categories of total clomiphene dosage and perinatal outcomes among singleton gestations in 21,004 fresh autologous in vitro fertilization embryo transfer cycles, 2004–2021.**

|  | **Total clomiphene citrate dose** | | | | | | | |
| --- | --- | --- | --- | --- | --- | --- | --- | --- |
|  | **<500 mg**  **N=4476** | | **500-749 mg**  **N=14241** | | **750-999 mg**  **N=1517** | | **≥1000 mg**  **N=770** | |
|  | N (%) | aRR^a^  (95% CI) | N (%) | aRR^a^  (95% CI) | N (%) | aRR^a^  (95% CI) | N (%) | aRR^a^  (95% CI) |
| Clinical pregnancy | 836 (18.7) | 1.00 | 2791 (19.6) | 1.02  (0.96-1.10) | 311 (20.5) | 1.01  (0.90-1.14) | 144 (18.7) | 0.93  (0.79-1.08) |
| Spontaneous abortion | 116 (2.6) | 1.00 | 502 (3.5) | 1.32  (1.07-1.62) | 59 (3.9) | 1.47  (1.08-2.00) | 17 (2.2) | 0.85  (0.51-1.40) |
| Stillbirth | -^b^ | 1.00 | 20 (0.1) | -^b^ | -^b^ | -^b^ | 0 | -^b^ |
| Live birth | 688 (15.4) | 1.00 | 2201 (15.5) | 0.98  (0.90-1.06) | 244 (16.1) | 0.94  (0.83-1.08) | 118 (15.3) | 0.90  (0.76-1.08) |

*Note*: Stillbirth outcome was too rare to estimate reliably relative risks. mg = milligrams; aRR = adjusted risk ratio; CI = confidence interval.

^a^ Adjusted for age, parity, number of prior ART cycles, infertility diagnosis, race/ethnicity (imputed), and body mass index (imputed).

^b^ Cell suppressed due to small numbers (1–4).
